# RESILIENCE AND PSYCHOLOGICAL DISTRESS IN GENETIC TESTING FOR ALZHEIMER’S DISEASE

**DOI:** 10.1093/geroni/igad104.3574

**Published:** 2023-12-21

**Authors:** Alicia Burgei

**Affiliations:** Tiffin University, Tiffin, Ohio, United States

## Abstract

This research project examines the relationship between resilience and psychological distress resulting from genetic testing for Alzheimer’s disease among individuals who are non-cognitively impaired. Preventative genetic testing is increasingly important to determine the likelihood of developing or passing on genetic disorders. However, receiving genetic information, especially regarding incurable diseases like Alzheimer’s disease, can lead to significant psychological distress. Resilience, the ability to cope with adversity and recover quickly, is considered a protective factor against psychological distress. The purpose of this study is to investigate whether higher levels of resilience are associated with lower psychological distress from genetic testing for Alzheimer’s disease. A cross-sectional survey was conducted online using the Impact of Genetic Testing for Alzheimer’s Disease (IGT-AD) scale and the Brief Resilience Scale (BRS), along with a question about family history of Alzheimer’s disease. The results indicated a significant negative correlation between resilience and psychological distress, suggesting that individuals with higher resilience experienced lower distress related to genetic testing. However, family history did not moderate the relationship, meaning the protective effect of resilience was consistent regardless of familial risk. These findings have implications for developing targeted support services and resilience-based interventions to help individuals cope with the emotional impact of genetic testing for Alzheimer’s disease. Further research could explore other potential protective factors and examine the long-term impact of genetic testing results on psychological well-being and behavior.

